# Modal Response Improvement of Periodic Lattice Materials with a Shear Modulus-Based FE Homogenized Model

**DOI:** 10.3390/ma17061314

**Published:** 2024-03-12

**Authors:** Tianheng Luo, Lizhe Wang, Fuyuan Liu, Min Chen, Ji Li

**Affiliations:** 1 School of Advanced Technology, Xi’an Jiaotong-Liverpool University, Suzhou 210053, China; 2 Institute of Orthopaedics & Musculoskeletal Science, Division of Surgery & Interventional Science, University College London, Royal National Orthopaedic Hospital, Stanmore, London HA7 4LP, UK; 3 School of Engineering, University of Liverpool, Liverpool L69 3BX, UK; 4 Key Laboratory of MEMS of the Ministry of Education, Southeast University, Nanjing 210096, China

**Keywords:** lattice structure, homogenization modeling, periodic boundary conditions (PBCs), modal analysis, anti-vibration capacity, finite element method (FEM)

## Abstract

Lattice materials are widely used in industries due to their designable capabilities of specific stiffness and energy absorption. However, evaluating the mechanical response of macroscopic lattice structures can be computationally expensive. Homogenization-based multi-scale analysis offers an efficient approach to address this issue. To achieve a simpler, while precise, homogenization, the authors proposed an equidistant segmentation (ES) method for the measurement of the effective shear modulus. In this method, the periodic boundary conditions (PBCs) are approximated by constraining the lateral displacement of nodes between parallel layers of periodic cells. The validations were applied to three typical lattice topologies: body-centered cubic (BCC) lattices, gyroid-, and primitive-triply periodic minimal surface (TPMS) lattices, to predict and compare their anti-vibration capacities. The results demonstrated the rationality and the promising precision of the multi-scale-based equivalent modal analysis through the proposed method and that it eliminated the geometric limitation of lattices with diverse frameworks. Overall, a higher anti-vibration capacity of TPMS was observed. In the study, the authors examined the influence of the relative densities on the balance between the anti-vibration capacity and loading capacity (per unit mass) of the TPMS topologies. Specifically, the unit mass of the TPMS with lower relative densities was able to resist higher frequencies, and the structures were dominated by the anti-vibration capacity. In contrast, a higher relative density is better when emphasizing the loading capacity. These findings may provide notable references to the designers and inform the selection of lattice materials for various industrial applications.

## 1. Introduction

Throughout nature, materials with a repeated cellular structure are ubiquitous and are found in a variety of carriers such as sponges, bamboo, and honeycombs. In 1982, Gibson et al. first convincingly studied these materials from both two-dimensional and three-dimensional perspectives, introducing their mechanical analysis [1,2]. Lattice materials, as one branch of the cellular ones, have been proven to have increasing research potential due to their great specific modulus, the capacity of energy absorption and anti-vibration, as well as the control of fluid flows and heat transfer [3,4]. Recent advances in additive manufacturing (AM) have been a driving force behind the production of lattice materials with high accuracy and complexity at a low cost, significantly expanding the depth and breadth of their studies. As a result, lattice materials have experienced an upward trend in application across the biomedical, aerospace, and automotive fields in the past ten years [5], where precision and custom design are essential.

The design in porosity contributes to the functionality of the lattice materials. The mechanical performances of lattice materials have been extensively investigated in the literature under statical and dynamic loading conditions, such as tension and compression, bending and buckling, transient energy absorption, as well as fatigue failure [3,5,6,7,8,9,10,11,12,13]. Conversely, relatively few studies have been conducted on modal analysis despite their existing application in the design of medical devices, architecture, and aerospace, in which anti-vibration capacity is crucial [14,15,16,17,18,19]. Additionally, a myriad of studies in the literature were solely established at a single scope: macroscopic ones without considering the periodic boundary conditions (PBCs), which limits their ability to accurately represent the behavior of global material. To address this, multi-scale modeling for periodic lattice materials is an efficient strategy. Their mesoscopic structures are constructed through the periodic arrangement of minimum unit cells along the various degree-of-freedoms (DoFs), allowing the extraction of the representative volume elements (RVEs). As defined, RVEs should possess the meso-structural characteristics of the materials and be able to anticipate mechanical behaviors to the global medium under macroscopic boundary conditions [20]. Researchers can formulate functional lattices at the RVE level and then validate them numerically and experimentally under PBCs to construct conformal macroscopic designs [18,19]. This especially benefits from the development of numerical computation methods, most notably the finite element method (FEM), which offers solutions without making use of simplifying assumptions commonly made during the analytical derivations [21]. By utilizing FEM, researchers can account for geometrical details and manufacturing imperfections more accurately, leading to more realistic results.

However, the benefits and issues are correlated during the macroscopic analysis. While direct computation at the macroscopic structure intuitively reflects the global mechanical response, it also leads to exponential growth in computational load with the extension of the RVEs and superposed loading conditions. In this context, the homogenization technique provides a balanced approach to performing reliable predictions with low consumption of time and resources. Over the decades, homogenization-based multi-scale analyzing methods have been developed and applied in various disciplines, ranging from physics to computer science [20].

The methods are generally classified into analytical-theory-based and numerical-computation-based. Analytical approaches, such as the beam theory approach, were initially employed to analyze the honeycombs by Gibson and Ashby, and then other researchers investigated further based on it [1,2,22,23]. Hohe et al. carried out an investigation through the strain energy equivalence, validly determining the behaviors of the macroscopic cellular sandwich cores fully based on their RVEs through the basic law of continuum mechanics [24]. For the cases that were not applicable to the classical continuum theory, Cosserat and Eringen proposed and revised the method when the structures are subjected to high strain gradients, such as crack tips, by considering additional microstructural joint rotation in addition to the translational displacements [25,26]. Elsayed et al. conducted an investigation on the Cauchy–Born hypothesis to approximate the solid-state physics to the solid mechanics of lattice structures and employed a Dummy Node Scheme to construct the nodal periodicity [27]. Another well-established method, which is also the basis of modern FE approaches, is asymptotic homogenization. It constrains the displacements of the opposite edges to guarantee the strain fields [28]. While asymptotic homogenization has its superiority, the biggest drawback is the huge computational cost, especially when it involves complex topologies.

In numerical homogenization, simple FE specimens can be easily homogenized for statically tensile or compressive conditions and modeled with equivalent mechanical properties (i.e., modulus, inertia, etc.). On the basis of the stiffness matrix of a material, the determinants include anisotropic/isotropic effective elastic modulus, transverse shear modulus, Poisson’s ratio, and density. Yet, the independent determination of the effective shear modulus, which is one of the key factors during the dynamic analysis, was much less discussed.

Yang et al. proposed a detailed numerical methodology for establishing the improved equivalent model of 2D corrugated sandwich structures to predict their dynamic response, which was of considerable reference value in numerical modeling [29]. Alwattar et al. expanded the numerical approach by integrating the neural networks (NNs), which effectively reduced the manual workload [30]. While their validations demonstrated the accuracy of the NNs, they did not adequately demonstrate the correctness of the effective shear modulus, as it did not affect the compression experiment results they conducted. Besides them, Panettieri et al. comprehensively carried out the homogenization of abundant types of strut-node topologies in modal analysis [21]. Rather than determining each effective property separately during the FE modeling, they computed the stiffness matrix of the RVEs. However, their homogenization errors were relatively large and had significant instability, from 5% to 15%. Apart from the above studies, which focused on the strut-node type lattice materials, recent researchers in the field of structural optimization are turning their attention to the bionic configurations due to their unique characteristics attributed to mathematically-driven designs. Abueidda et al. and Spear et al. carried out the experimental and FE investigations on a series of types of triply periodic minimal surfaces (TPMS) configuration and found that superior mechanical capacities were detected from the Gyroid-TPMS structure [31,32]. Despite this, bionic structures still lack investigation in dynamic conditions.

The present work aimed to propose an improved FE homogenization method with stability and low errors to predict the modal response of the 3D repeated lattice RVEs. A new idea to realize PBC in the shear direction that is simpler yet more accurate than using constraint equations has been developed. Moreover, the study investigated the sensitivity of the RVE size (which refers to the number of minimum unit cells in one selected RVE) to acquire the threshold dimension of RVEs for ideal outcomes. The effectiveness of the method was demonstrated by comparing undamped natural frequencies and corresponding mode shapes of the original body-centered cubic (BCC) lattice RVEs and the homogeneous models through free-end modal analysis. Subsequently, the validations were extended to the macroscopic scale to predict the anti-vibration capacities. In addition to the strut-node topology, the study was also carried out on the mathematically driven gyroid- and primitive-TPMS topologies. By bringing the relative density into the geometric variables, the investigation presented the comparisons of the anti-vibration capacity and addressed several conclusions regarding the relationship between the relative densities, anti-vibration capacity, and the loading capacity, which is expected to be useful for industrial usage.

## 2. Homogenization Modeling

### 2.1. General Periodic Boundary Conditions

The displacement field of the macroscopic lattice structure with heterogeneous material can be evaluated from the PBCs-based mesoscopic unit cell [33,34], which was expressed as:

(1) $$u_{i}={\bar{\varepsilon }}_{ik}x_{k}+u_{i}^{*},$$

where ${\bar{\varepsilon }}_{ik}$ is the average strain for the unit cell, $x_{k}$ is the coordinate for any node in this unit cell, and $u_{i}^{*}$ is a periodic displacement correction depending on the global loading condition, being assigned with the same value on the corresponding parallel planes. The PBC theory has been maturely discovered in the literature, and it falls out of the scope of this project; therefore, detailed discussions on the theory will not be provided in this paper.

**Figure 1:**
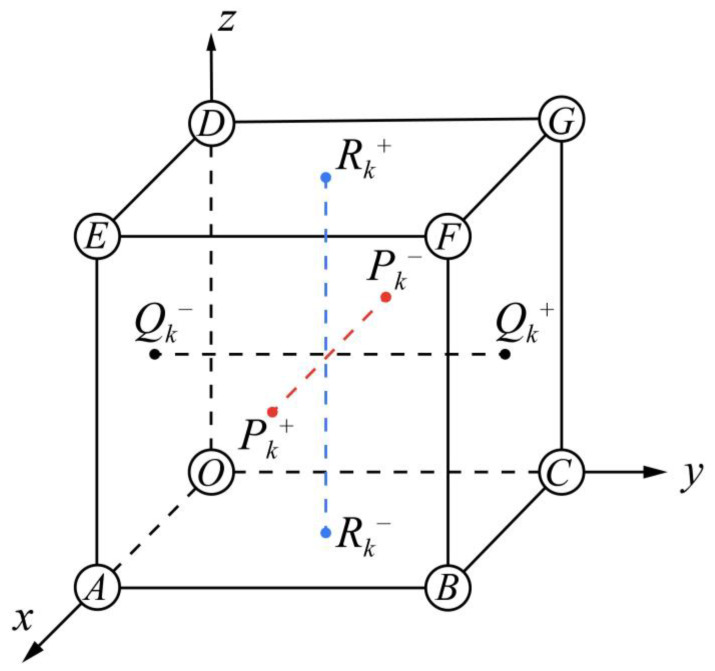
Diagram of a general 3D RVE with nodal pairs.

To implement PBCs for this scenario in commercial FE software (ANSYS 2020), the DoFs of mesh nodal pairs on the corresponding parallel planes are typically coupled. Figure 1 shows a general 3D RVE with three sets of nodal pairs: $P_{k}^{+}$–$P_{k}^{-}$, $Q_{k}^{+}$–$Q_{k}^{-}$ and $R_{k}^{+}$–$R_{k}^{-}$, where the notation *k* refers to the coordinates along the *x*-, *y*-, and *z*-axis. These nodal pairs represent all the one-to-one normal-parallel nodes on the periodic boundary planes in the positive and negative directions of a specific coordinate axis.

To enforce the rigid displacement constraint, a corner node *O* located at the origin is fixed in all 6 DoFs. In the three pairs of parallel planes, those with x=0, y=0, and z=0 are defined as the subordinate planes, and their opposite sides are then the principal planes. During the deformation, the displacement difference between all the nodes on the principal plane that are orthogonally along the *x*-axis, denoted by $P_{k}^{+}$, and those on the subordinate plane, $P_{k}^{-}$, should have the same magnitude as that between the nodes *O* and *A*. Hence, the nodal constraint for $P_{k}^{+}$ and $P_{k}^{-}$ orthogonally along the *x*-axis is implemented through

(2) $$\left\{\begin{array}{c} U_{P_{k}^{+}}-U_{P_{k}^{-}}-U_{A}+U_{O}=0\\ V_{P_{k}^{+}}-V_{P_{k}^{-}}-V_{A}+V_{O}=0\\ W_{P_{k}^{+}}-W_{P_{k}^{-}}-W_{A}+W_{O}=0 \end{array}\right.,$$

where *U*, *V*, and *W* denote the displacement along the *x*-, *y*-, and *z*-axis, respectively. Similarly, the displacement differences orthogonally along the *y*- and *z*-axis are the ones between nodes *O* and *C* as well as *O* and *D*, respectively. Appropriate settings and coding in FE software can ensure reasonable implementation of the above PBCs.

As discussed above, the implementation of PBCs hinges on the one-to-one corresponding nodal pairs for all three principal and subordinate planes, which necessitates identical meshing on the planes in all three dimensions simultaneously. Unlike solid cubes, which can be easily meshed through the hexahedral elements, porous and/or curved topologies are often meshed through the tetrahedron elements to reduce intensive labor. The nodal distributions are much less regularly arranged, so they may fail to be identical on all the opposite planes due to the merging of nodes on the shared edges. This problem is exacerbated for bionic and mathematically driven topologies that possibly lack symmetry about the mid-plane. To address this challenge, the author proposes a new approach that constrains the deforming trajectory, rather than just matching nodal pairs, to enable PBCs to be applied for all three dimensions simultaneously. This approach obviates the need for generating identical mesh nodes on opposite planes and can be employed for any symmetric or asymmetric meshing results of the topologies as long as the RVE is geometrically repeatable in the material. This novel approach offers a more convenient gateway to determine the effective shear modulus for repeated strut-node and bionic topologies, facilitating anti-vibration analysis through homogeneous models. Detailed discussions and implementations of this approach are presented in Section 2.3.

### 2.2. Equivalent Properties under Tensile Force

**Figure 2:**
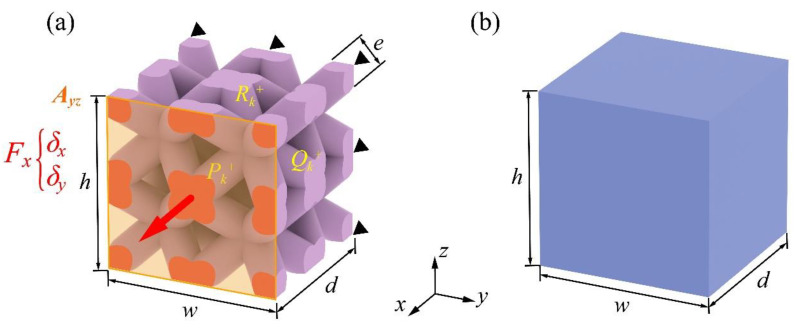
Illustration of (**a**) periodic lattice material, and (**b**) homogeneous model.

The effective parameters of the orthotropic homogenization encompass the elastic modulus $E_{i}$, the shear modulus $G_{ij},$ and in-plane Poisson’s ratio $\nu_{ij}$, which can be determined using Hooke’s law through six sets of independent stress fields applied on the RVE. In this paper, the methodology will be conducted through the BCC structure with a geometric symmetry orthogonal in *x*-, *y*-, and *z*-directions. The filling material itself is isotropic, resulting in a quasi-isotropic mechanical behavior for the BCC RVE. This enables the elastic modulus, Poisson’s ratio, and shear modulus have, respectively, the same value in all three orthogonal directions. Thus it should be noted that the stiffness matrix obtained through this method is applicable only to isotropic or orthotropic materials. This means it is limited to structures that exhibit three mutually perpendicular planes and a 90-degree rotation with respect to those planes. Therefore, for anisotropic materials, the physical behaviors in all directions cannot be accurately predicted using this method. To investigate the homogenization modeling while considering how the RVE size will affect the response of the modeling, a series of RVEs comprised of an incremental number of minimum unit cells were adopted to establish the FE models, using the FE software (ANSYS 2020). Among the geometric parameters, *n* denotes the number of unit cells in each dimension (i.e., n×n×n BCC configuration). Figure 2 illustrates the lattice and homogeneous FE models with an equivalent measured volume in a Cartesian coordinate system. Here, *w*, *d*, *h*, and *e* represent the width, depth, height, and rod diameter of the lattice RVE, respectively.

The determination of tensile elastic modulus $E_{i}$, Poisson’s ratio $\nu_{ij}$, and equivalent mass density $\rho_{eq}$ was conducted through the FE models shown in Figure 2a, with n=2 serving as an example. To enforce PBCs, nodal coupling was defined for all the mesh nodes in the three principal planes along the deformed directions, respectively. The RVE was fixed on the subordinate plane along the *x*-axis to prevent rigid displacement, while on its opposite side, an active displacement was applied (marked in red). It is worth noting that the RVE was allowed to contract along the −*y*-axis and the −*z*-axis, depending on its Poisson’s ratio. Consequently, the effective elastic modulus and Poisson’s ratio were calculated using Equation (3) and Equation (4), respectively:

(3) Ex=σxεx=FxAyzδxlx=Fxwhδxd

(4) νxy=−εyεx=−δylyδxlx=−δywδxd,

where $F_{x}$ is the reaction force on the $P_{k}^{+}$ plane and $l_{i}\;(i=x,y)$ is the distance between the two parallel planes. The equivalent mass density is defined as the ratio of the mass of the lattice RVE and its measured size, calculated using:

(5) $$\rho_{eq}=\frac{m_{RVE}}{V_{measured}}.$$

### 2.3. Equivalent Transverse Shear Modulus

Based on the continuous mechanics of homogeneous materials, the shear modulus, *G*, of the isotropic material usually fulfills the relationship with its elastic modulus and Poisson’s ratio, given as:

(6) $$G=\frac{E}{2(1+\nu )}$$

However, this equation cannot be applied to lattice materials with isotropic raw materials due to their heterogeneous constructions and poriferous configurations. To homogenize these materials for modal analysis, it is hence crucial to independently determine the effective shear modulus. Besides the current method by the constraint equations on the nodal pairs, as reported by Xue et al., another potential approach to achieve PBCs is proposed in this paper. This method involves forcibly restricting the internal deformation trajectories and is referred to as the equidistant segmentation (ES) method.

**Figure 3:**
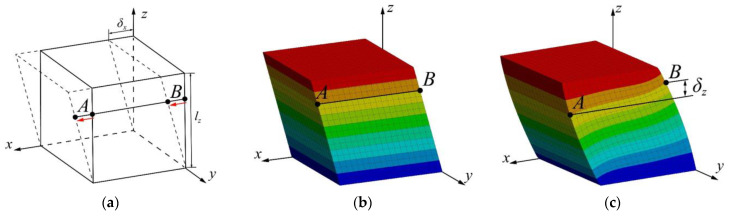
Illustration of (**a**) pure shearing strain from undeformed shape, (**b**) FE shearing deformation with ES method, and (**c**) FE shearing deformation without ES method.

To explain the concept, the author used a general cubic structure to represent the lattice RVE for a more intuitive illustration, as the cube has larger flat areas to display the deformation distribution. Figure 3a illustrates an undeformed model subjected to a pure shearing strain where $\delta_{x}$ is the displacement along the shearing direction and $l_{z}$ is the initial size of the model. The FE result of continuity expects what is illustrated in Figure 3b. In this figure, the periodicity of its physical field is presented as an instance. It can be observed that, from the perspective of the overall deformed shape, identical RVEs can be duplicated next to each other to fulfill the periodicity and transmit the mechanical properties. Additionally, internal deformation trajectories uniformly distribute as distance increases from the shearing load. Nodes *A* and *B* denote any two nodes with the same original *z*-location, and they should remain at the same *z*-location after shearing. Otherwise, the shearing outcome will result in a loss of periodicity in the shearing direction and non-uniform internal deformation, as seen in Figure 3c. Nodes *A* and *B*, in this case, have a height difference, $\delta_{z}$. To correct this, nodal displacement at each layer along the *z*-axis can be mechanically constrained to forcibly prevent the nodes *A* and *B* from displacing by $\delta_{z}$.

**Figure 4:**
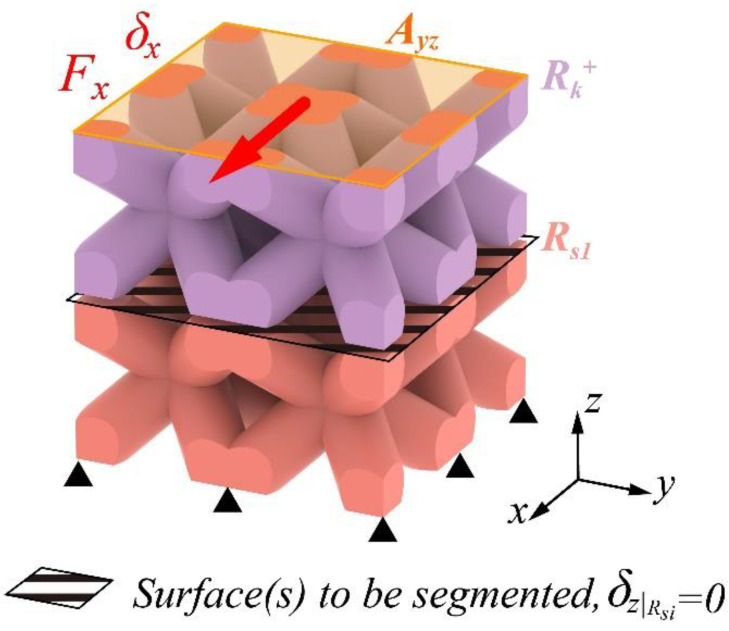
Illustration of the equidistant segmentation of the FE model for shear strain, *n* = 2.

Based on the above principle, Figure 4 illustrates the application of the ES method on the lattice RVE with n=2. In this method, the RVE is segmented along the transverse planes parallel to the displaced layer(s). These planes should always be where the minimum unit cells intersect, as shown in the shaded surface(s) of the figure. The displacement of the in-plane mesh nodes, $R_{si}(i=1,2,\ldots ,n-1\;\mathrm{and}\;n>1)$, on the segmented layers is coupled in the orthogonal axis (*z*-axis in this case). Note that through segmentation, the meshing is conducted separately for each layer of the RVE, but the nodes on the boundary faces should be merged to transfer the stress. In the FE model in Figure 4, an active transverse displacement $\delta_{x}$ was applied on the *+z* plane so the −*z* plane should be fixed. Under the above settings, the effective shear modulus was calculated using the equation:

(7) Gxz=τxzγxz=FxAxyδxlz=Fxwdδxh.

Section 2.3 reveals a major difference between the traditional method (Section 2.1) and the ES method. Essentially, the traditional approach applies the PBCs on the outer faces of periodic cells, which requires highly symmetric meshing results on the two outer surfaces along each axis so the constraint equations can enforce the displacement difference between nodal pairs (Figure 3 refers to nodal pairs on the opposite outer faces in the *zy*-plane). In cases where the shapes of the cells are complex, such as TPMS, achieving identical meshing results on corresponding surfaces along all axes becomes challenging, making it already labor-intensive during the pre-processing to realize PBCs in all three dimensions simultaneously.

Contrarily, the ES method ignores the meshing results, thereby significantly reducing the labor. Primarily, the segmentation between the minimum periodic cells ensures the identical shapes of all sub-layers. Subsequently, the nodal coupling guarantees that all sub-layers between the periodic cells perform the same physical behavior—pure transverse shearing—so that the entire structure also behaves as pure transverse shearing. Consequently, only the displacements of nodes on these parallel layers (refers to all the nodes like node A and B on each layer in the *xy*-plane in Figure 3) are constrained in the orthogonal direction (refers to the *z*-direction in Figure 3).

## 3. Results and Discussion

### 3.1. Mesoscopic FE Validation

The anti-vibration capacity can be evaluated by performing free-end modal analysis, which determines the undamped natural frequencies of the structures in a series of vibrating modes. Of all the natural frequencies, the 1st mode natural frequency is particularly important as it represents the lowest frequency at which the structure resonates. A higher 1st mode natural frequency indicates a higher threshold of the structure to reach its failure, indicating a stronger capacity to withstand the vibration. In this section, the authors examined the effectiveness of the homogeneous model under dynamic conditions by performing modal analysis on both the BCC RVEs and their corresponding homogeneous models. Naturally, the construction of the homogeneous models deployed ES-based strategy (Section 2.2 and Section 2.3) to recover all the necessary mechanical properties in a modal analysis. In the beginning, variations in the numbers and size of meshing elements were discussed to determine the converged preconditions of homogenization.

**Figure 5:**
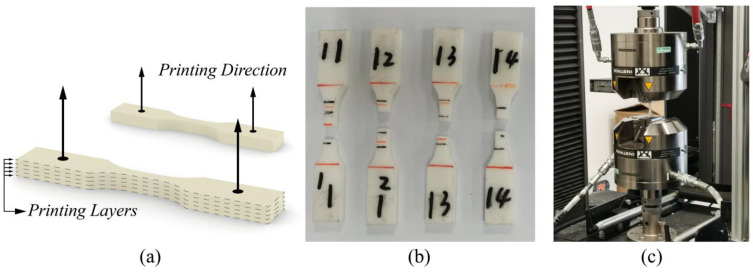
Material test: (**a**) illustration of the printing direction, (**b**) printed specimens, and (**c**) tensile test using the Instron 5982 static tension machine.

The raw material for all the FE simulations was isotropic resin manufactured by the Formlabs-Form3 SLA 3D printer with a printing direction shown in Figure 5a and a precision of 25 μm. The post-process involved washing with isopropanol for 10 min and curing at 60 degrees Celsius for 60 min. The average material properties in Table 1 were measured following the tensile test standard (ASTM D638-14 [35]) using the Instron 5982 static tension machine (as shown in Figure 5b,c).

**Figure 6:**
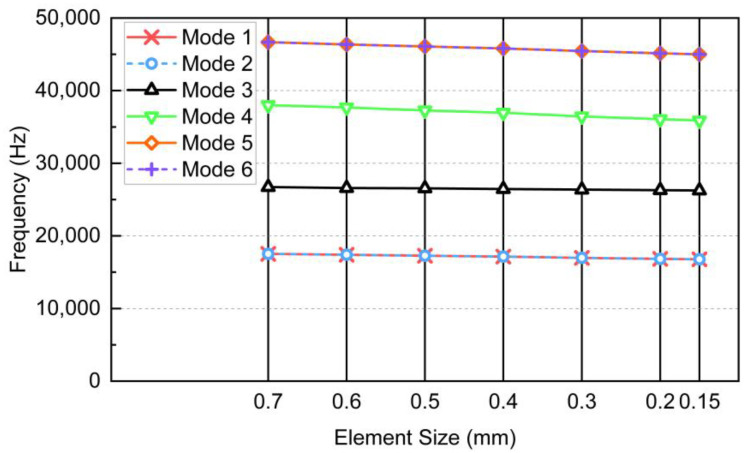
Mesh convergence study for the FE model.

**Figure 7:**
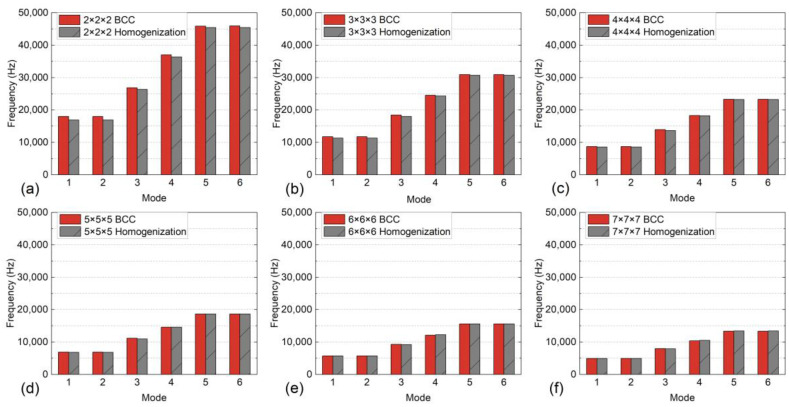
Variation of the natural frequencies with respect to *n*: (**a**) *n* = 2, (**b**) *n* = 3, (**c**) *n* = 4, (**d**) *n* = 5, (**e**) *n* = 6, and (**f**) *n* = 7.

**Figure 8:**
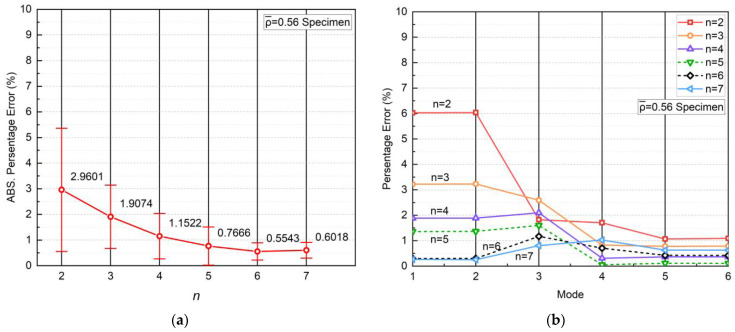
Variation of absolute errors between lattice RVEs and homogeneous models with respect to n in the view of (**a**) average errors of six modes and (**b**) errors of each mode.

A mesh convergence study was carried out on the FE models. The authors selected a minimum BCC RVE with a size of 3 mm in each dimension. Its dimensions are listed in Table 2. As depicted in Figure 6, the structure exhibits low sensitivity to the element size. Although a greater number of elements results in slightly finer results for the analysis, it has a negligible effect on the conclusion in this case, but significantly increases computation. Therefore, the mesh size of 0.3 mm was chosen for the subsequent studies, taking into account the computational cost. It is noteworthy these BCC RVEs are identical and symmetrical in all three axes. The vibrating axes in mode 1, 2, 5 and 6, respectively, are perpendicular to each other. Hence, their mode shapes that have similar natural frequencies are mirrored about the midlines of the two vibrating axes, meaning they have perpendicular directions. Furthermore, the size effect of the RVE was examined by plotting the natural frequencies as *n* increases from 2 to 7, as shown in Figure 7. Collectively, an excellent agreement was found before and after homogenization. The average absolute errors of the 6 modes, as shown in Figure 8a, indicated the maximum error was 2.9601% for n=2. In previous studies, the authors observed that the elastic modulus of a BCC topology typically converges at n=3 under uniaxial tensile test. However, when the dynamic loading condition is introduced, more mechanical property variables require a greater size to converge. As *n* increased, the average error gradually declined until *n* reached 5, when the error tended to converge at a value of between 0.7666% and 0.5543%. This manifests the size of it no longer has a significant effect on the average dynamic response once the RVEs are composed of no less than 5 minimum unit cells in each dimension (i.e., 5×5×5). Moreover, the homogeneous natural frequencies tend to gradually exceed those of the original lattices as *n* increases, which is another influence of the size.

**Figure 9:**
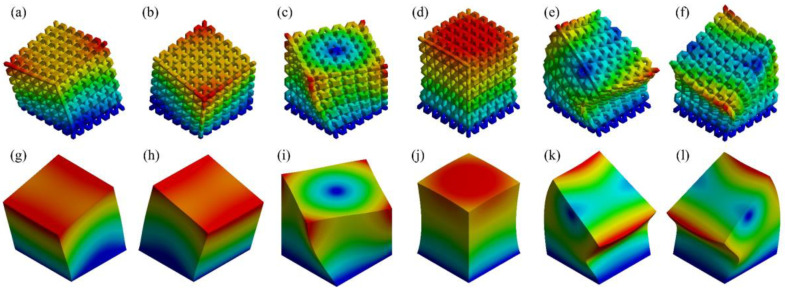
The first six modes’ mode shapes (0.005 × scale) of: (**a**–**f**) 6×6×6 BCC RVEs, and (**g**–**l**) homogeneous models.

Besides, a mode-based comparison in Figure 8b reveals the alteration of *n* primarily affects the natural frequencies of the first three modes. Although the average absolute error in the case of n=5 is analogous to that of n=6 and 7, n=5 case has errors in modes 1~3 significantly greater than errors in modes 4~6. This implies that despite n=5 achieved an ideal overall outcome, n≥6 is still preferred for a more accurate equivalence of each mode respectively. Regarding the mode shapes, the results deducted from the actual 6×6×6 RVE and its homogeneous model demonstrate excellent consistency, as illustrated in Figure 9. For shapes that involve bending (modes 1, 2, 5, and 6), the deformation distributions marginally vary on the corners of the structures. This is because homogenization modeling focuses solely on the global response of the structures while ignoring their local deformation determined by the inner configuration. As a result, the porosity of the lattice structure leads to less local stiffness than that of the cubes. Nevertheless, the authors acknowledge that the above conclusions may not be universally applicable since the size effect was only studied on the BCC topology in this study. Materials with other topologies may exhibit different sensitivities to sizes. To maintain the consistency of the study and eliminate the possible influence of the RVE size, the following studies were all carried out based on 6×6×6 minimum RVE number.

In this section, the influences of the effective elastic modulus E, Poisson’s ratio ν, and shear modulus G on the natural frequencies of the BCC lattice were investigated. The aim was to observe the correlations/trends between each of those mechanical properties and the modal outcome, respectively. Having established the correlations, we were able to carry out the sensitivity analysis of these mechanical properties to obtain the extent of their effect. This helped to judge and appreciate the significance of the ES method.

**Figure 10:**
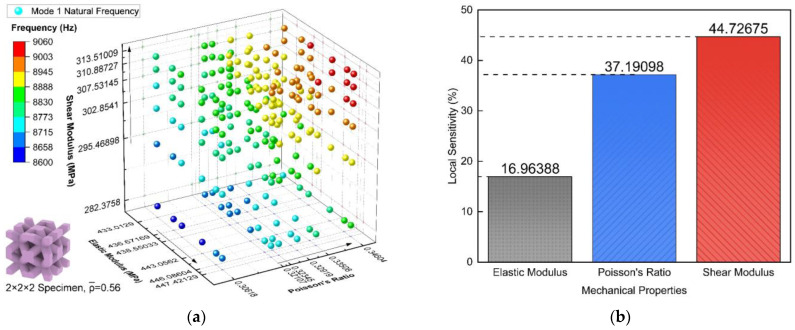
The influence of the mechanical properties on the natural frequencies: (**a**) 1st mode natural frequency under growing effective parameters, and (**b**) sensitivity analysis.

The study randomly selected a range on the basis of the true values for the elastic, shear modulus, and Poisson’s ratio, respectively (the principle of conducting a sensitivity analysis). These changing mechanical properties were suppositional but based on the true values carried out from a 2×2×2 BCC RVE with a relative density of 0.56. The relative density remained unchanged for the variable control. Figure 10a displays the 1st mode natural frequency of a n=2 RVE changed as the elastic modulus varied from 433.01 MPa to 447.42 MPa, the Poisson’s ratio varied from 0.31 to 0.35, and the shear modulus varied from 282.38 MPa to 313.51 MPa. The results indicate an increase in any of these effective parameters raised the natural frequencies of the RVE. Subsequently, the sensitivity analysis result is shown in Figure 10b. It states all the parameters had positive correlations with the natural frequencies, but the shear modulus exerts the most influence. This makes the shear modulus the dominant factor in the anti-vibration capacity of the structure and naturally supports the significance of the ES method.

**Figure 11:**
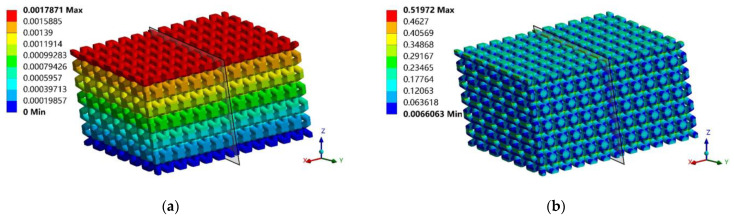
The periodic mechanical behavior in the transverse shearing direction (300× scale): (**a**) deformation and (**b**) Von-Mises stress.

To intuitively present the periodic continuity achieved by the ES method, the authors duplicated a deformed n=6 RVE under shearing force next to another one in Figure 11. Both the physical field and stress field achieve proper periodicities in the transverse shearing direction (along the *x*-axis), as expected from Figure 3b. This confirms that the effective shear modulus plays the correct role during the homogenization process.

### 3.2. Macroscopic FE Validation

**Figure 12:**
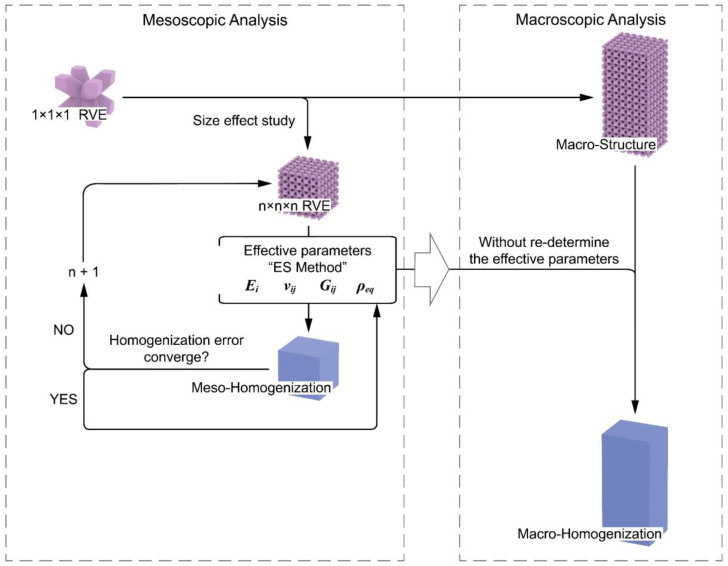
The process that uses the homogenization model to perform the multi-scale modal analysis.

**Figure 13:**
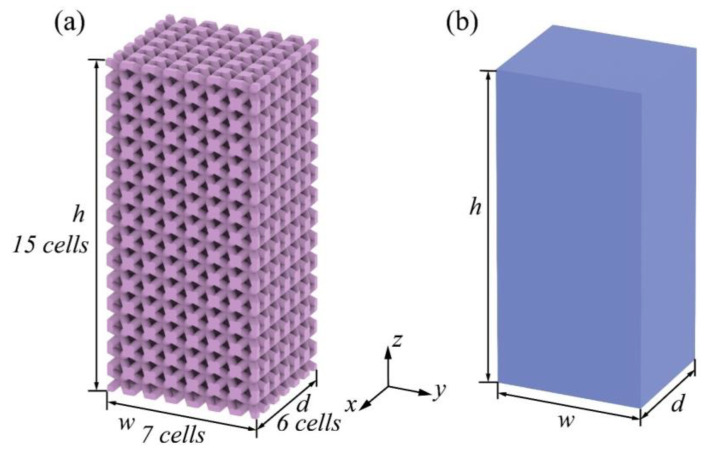
Illustration of a macroscopic BCC structure: (**a**) 6×7×15 FE model, and (**b**) homogeneous model.

This section presents the validation of the homogenization approach at a macroscopic scale. The process chart in Figure 12 demonstrates the procedure of the multi-scale analysis. The procedure begins with investigating the size effect of the RVEs. The ES method is employed to determine four effective parameters of the lattice, which are then assigned to the mesoscopic homogenization model. The minimum number of periodic cells is gradually increased until the homogenization error converges, ensuring that the parameters obtained from this size of RVE are sufficiently accurate for homogenization. **These parameters are then directly used for homogenizing any larger structures in the macro-scale without the need for a re-determination.** This process guarantees an adequate homogenization for the macroscopic structures while requiring only minimal effort from the mesoscopic systems. To distinguish between meso- and macro-scopes, the selected macroscopic structure should ideally be much larger than 6×6×6 (such as 60×70×150). Unfortunately, the computation greatly exceeded the acceptable computing capability, making it impractical to analyze such a large structure. A 6×7×15 BCC structure was finally selected as a macroscopic specimen. Its FE and homogeneous models are illustrated in Figure 13. Its dimensions change as in Table 3 where the $\bar{\rho }$ is the relative density ($\bar{\rho }=V_{solid}/{(V}_{solid}+V_{void})$). The homogenized model was assigned with the same effective parameters ($E_{i}$, $G_{ij}$, $\nu_{ij}$, $\rho_{eq}$) deducted from the 6×6×6 RVE in Section 3.1.

**Figure 14:**
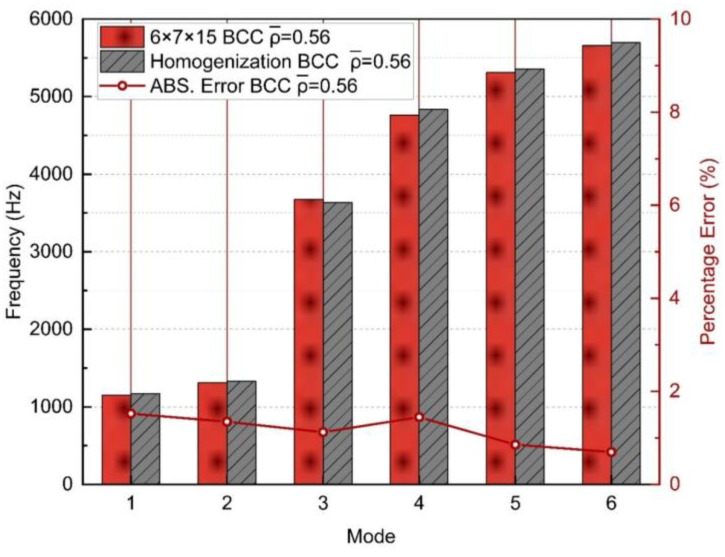
The first six modes of natural frequencies of the 6×7×15 lattice structure.

**Figure 15:**
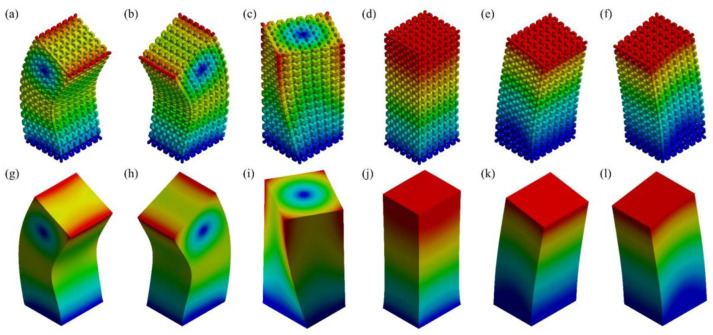
First six modes mode shapes (0.01 × scale) of: (**a**–**f**) 6×7×15 BCC RVEs, and (**g**–**l**) homogeneous models.

This validation was also through a free-end modal analysis and the first six modes of natural frequencies are presented in Figure 14 and Table 4 with absolute errors. Figure 15 displays the corresponding vibrating shapes. The results show a fine agreement between the actual lattice and the homogenized model with an average absolute error of 1.1640%. The maximum absolute error (1.5205%) occure for the 1st mode, and a minimum error (0.6950%) is observed for the 6th mode. While the errors remain small and distributed within a narrow range, it was noted that they were higher than those obtained with the 6×6×6 RVE, indicating that the proposed method was not perfect. Nevertheless, the results support the validity of the ES method for macroscopic structures and confirm that the use of an RVE with n≥6 leads to more stable results for all modes. Different from the size effect study in Section 3.1, the current effective mechanical properties were not re-obtained from a 6×7×15 RVE but 6×6×6. The errors can stack when the macroscopic size expands, so it is important to note that the errors may increase as the scale of the macroscopic structure grows.

### 3.3. Bionic Topologies Implementation

**Figure 16:**
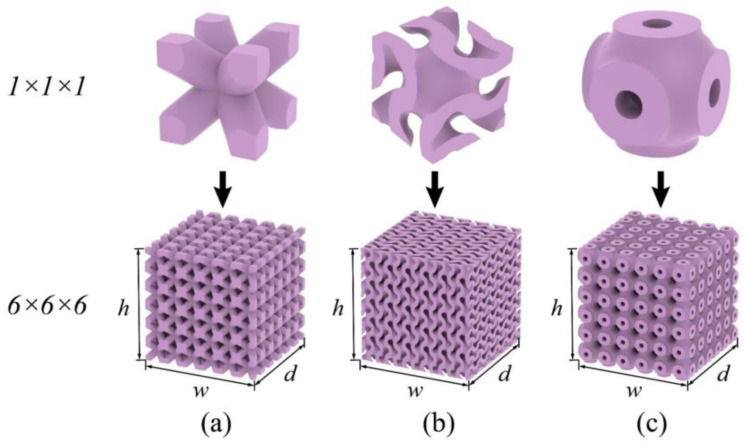
Illustration of the 1×1×1 and 6×6×6 topologies with relative density $\bar{\rho }=0.56$: (**a**) BCC, (**b**) gyroid-TPMS, and (**c**) primitive-TPMS.

To investigate the anti-vibration capacities of their bionic structures and the effectiveness of the ES method on them, vibration analysis was also conducted on gyroid- and primitive-TPMS cellular structures and their homogeneous models. The gyroid- and primitive-TPMS topologies here were generated from CAD software (Rhino 7) with the Grasshopper plugin through the mathematical expression, respectively, as:

(8) cos mx sin my+cos mysin mz+cos mzsin mx=0,

(9) cos mx+cos my+cos mz=0,

where m denotes the periodicity scaling factor that determines the number of unit cells repeated along the axes. The FE models of 1×1×1 and 6×6×6 BCC, G- and P-TPMS are with the same relative densities, illustrated in Figure 16a–c. Their dimensions are listed in Table 5.

**Figure 17:**
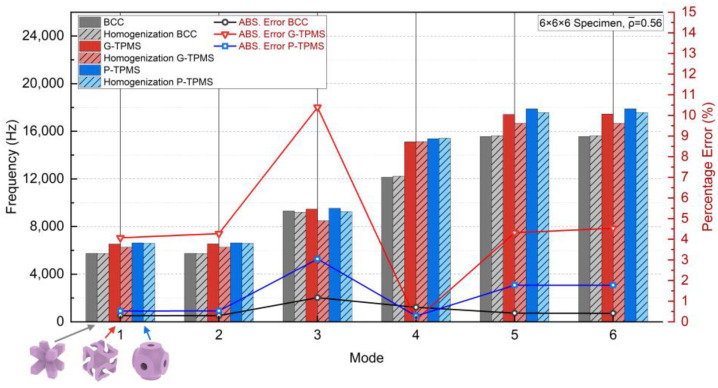
The first six modes’ natural frequencies of the three macroscopic BCC and TPMS topologies.

**Figure 18:**
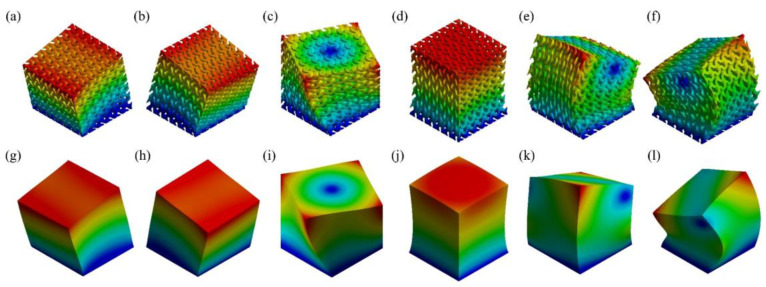
The first six modes’ mode shapes (0.005 × scale) of (**a**–**f**) 6×6×6 G-TPMS structures, and (**g**–**l**) homogeneous models.

The $E_{i}$, $G_{ij}$, $\nu_{ij}$, and $\rho_{eq}$ were also determined using the ES-based method. To compare their anti-vibration capacity and validate the precision of the homogenization, the 6×6×6 models were subjected to modal analyses. Figure 17 summarizes the first six modes of natural frequencies with absolute errors following the same procedure in the previous cases. Of the three topologies considered, the BCC one exhibited the lowest natural frequencies for all six modes, indicating that the TPMS structures had better anti-vibration capacity under the same weight. While the P-TPMS topology only slightly outperformed G-TPMS one in natural frequencies, the homogenization accuracy was distinctly more obvious, with an average error of 4.6170% in G-TPMS and 1.3241% in P-TPMS. Both exhibited the highest error in mode 3, which was dominated by the shear modulus during torsional deformation. On the contrary, the results between the BCC lattice and its homogenized model showed a much better consistency with an average error of 0.5543%. This implies the accuracy of the effective shear modulus decreased with the increasing geometric complexity. Nevertheless, the results demonstrate the feasibility of applying this approach to bionic structures, as demonstrated by the mode shapes of the G-TPMS topology in Figure 18.

**Figure 19:**
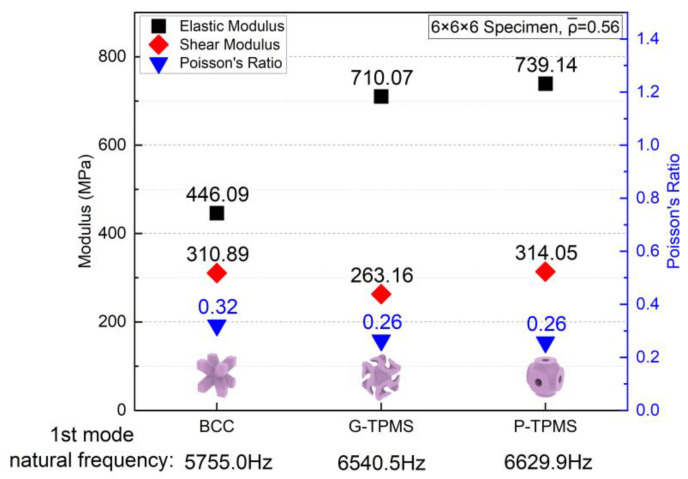
Effective modulus and Poisson’s ratio of the BCC and TPMS topologies.

Figure 19 presents a summary of the E, G, and ν for the three structures topologies. At the same relative density, both TPMS structures perform significantly greater elastic modulus than the BCC structure, which results in lower Poisson ratios. The shear modulus of the G-TPMS topology was the smallest among the three, although their shear modulus was generally similar. Therefore, the BCC structure exhibited the worst anti-vibration capacity with a much lower 1st mode natural frequency. Comparing the G- and P-TPMS ones, the latter performed better in terms of both elastic and shear modulus, resulting in the highest 1st mode natural frequency of 6629.9 Hz, and, therefore, the best anti-vibration capacity of all three structures.

To investigate the correlation between relative densities and the anti-vibration capacity of the bionic lattices, further parametric studies were carried out on the G- and P-TPMS topologies. In the previous sections, all structures were modeled and tested with the relative density $\bar{\rho }=0.56$. In this section, a series of TPMS lattices with the relative densities $\bar{\rho }=0.10,\;0.19,\;0.30,\;0.40,\;0.50$ were additionally examined. They were constructed into 6×6×6 mesoscopic RVEs with a unit cell size of 3 mm, as the same in Figure 16, for homogenization and free-end modal analysis. As the relative density decreases to $\bar{\rho }=0.10$, the wall thickness of the G-TPMS is approaching 0.05 mm, causing the FE software to fail to compute the effective parameters because the cross-sectional area to apply the load is too small. Hence, there will be no homogeneous model for it. Nonetheless, from the experience of the author, the thickness of 0.05 mm exceeded the precision limit for most types of AM machines, so in the case of $\bar{\rho }=0.10$ in a 3 mm unit cell may not have a practical value. Yet, once the size of the unit cells is increased, possible solutions for the effective parameters and manufacturing will also be available.

**Figure 20:**
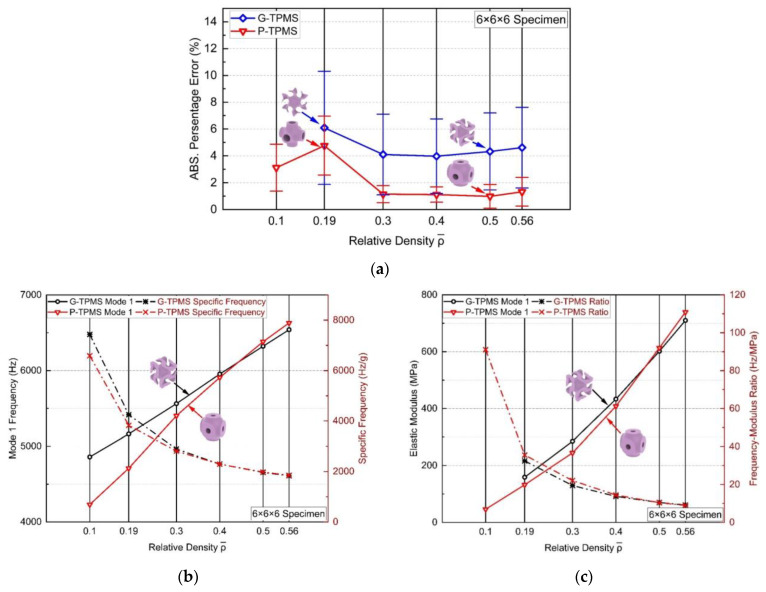
Influence of the relative densities on the modal results of the (**a**) errors of the homogenization, (**b**) 1st mode natural frequencies and corresponding specific frequencies, and (**c**) elastic modulus and corresponding specific modulus.

Figure 20a presents the errors between the original lattices and their homogeneous models. As the relative density increased above 0.3, the average errors stabilized at 1.1426% for P-TPMS and 4.2563% for G-TPMS. However, the errors from both topologies significantly rose once the relative densities were below 0.3. The meshing size was converged, so this shares the reason above. As the wall thickness approached extremely small values, the TPMS structure (and even the BCC structures as well) started to involve prominent nonlinear responses on the walls (or struts), such as buckling and torsion, which were not accounted for in the simple cube of the homogenized model. This caused larger errors in homogenization, and the FE software predicts unauthentically. Additionally, compared with the errors from the BCC topologies in Figure 8a, the overall errors increased as the geometric complexity grew.

To compare the anti-vibration capacity of the TPMS structures, their 1st mode natural frequencies were extracted from the modal analysis. The authors found that while the natural frequencies increase as the densities increase, the frequency accelerates much slower than that of the relative density. To quantify this feature, the authors have introduced a specific frequency, denoted as F, which describes the relative anti-vibration capacity, i.e., frequency withstood per unit mass (in Hz/g):

(10) $$F=\frac{f_{1}}{m}$$

where $f_{1}$ denotes the 1st mode natural frequency and m denotes the mass of the structure. Figure 20b summarizes the 1st mode natural frequencies and corresponding specific frequencies. The results indicate that the G-TPMS structure has a higher anti-vibration capacity at a low relative density, whereas the P-TPMS one surpasses it when the relative density exceeds approximately 0.45. Additionally, for both topologies, the specific frequencies decrease with the growth of the relative density, implying that a lower relative density may result in a more cost-effective structure (the minimum weight to withstand the highest frequency). When designers have a specific benchmark for the required anti-vibration capacity in their applications, they are encouraged to choose a lower relative density to achieve a lighter weight. Another relevant factor to consider when designing with TPMS structures is the loading capacity, which can be measured by the elastic modulus. A higher elastic modulus signifies greater tensile or compressive stiffness, and this means the material can bear a higher load (with the same loading area) before it starts to yield, under the same stress. Figure 20c shows the elastic modulus of the topologies and the frequency-to-elastic modulus ratio, R, defined as:

(11) $$R=\frac{f_{1}}{E}.$$

The results indicate the G-TPMS one exhibits a superior relative load capacity when the relative density is approximately 0.45 or lower, whereas the P-TPMS one outperforms it thereafter.

Furthermore, the combinatory view of Figure 20b,c contributes to the balance and bias between the capacities. During selection, a lower relative density is preferred if the designer aims to emphasize the anti-vibration capacity (anti-vibration dominant), whereas a higher relative density is preferable if the focus is on improving the loading capacity (loading dominant). Besides, both specific frequencies and the frequency-modulus ratio tend to converge at higher relative densities, indicating that designing topologies that exceed a certain high relative density may result in marginal effect and cause a wastage of weight and material. These quantitative conclusions are specific to the case examined in this study, but they may provide useful insights for designers in their industrial applications.

## 4. Conclusions

In this paper, the authors introduced an equidistant segmentation (ES) method-based PBCs approach for investigating the homogenization in modal analysis of lattice materials across multiple scales. The effectiveness of the method was validated by examining the natural frequencies and mode shapes in modal analysis with a series of BCC lattice RVEs. The study also demonstrated the wide applicability of the ES method by conducting extensive studies on gyroid- and primitive-TPMS structures. Based on the findings, several crucial conclusions were addressed below:

(1) Based on the proposed ES method, the deformation and stress field results showed accurate PBC in all directions when the effective shear modulus was determined. The results of the free-end modal analysis demonstrated excellent and stable coherence between the actual BCC lattice structures and their homogenization models under both mesoscopic and macroscopic conditions. Particularly, it is revealed that for the BCC lattice, the natural frequencies of the first three modes were more sensitive to the variation of size. The overall errors decreased as the RVE size grew and tended to converge after a size of 5×5×5. However, RVE sizes no less than 6×6×6 performed errors with less deviation for all modes, with errors of approximately 0.5% to 1%, which are lower than that of the previous studies in the literature [21].
(2) The effectiveness of the proposed ES method was demonstrated through its successful application to the parameterized bionic lattice configurations (gyroid- and primitive-TPMS) with various relative densities. It proved to be with high accuracy, as indicated by the converges errors of 4% and 1%, respectively, for the G- and P-TPMS. However, when the relative density was too small, the geometric influence tended to be too significant to fail the computation, resulting in decreased accuracy of the homogenization.
(3) Furthermore, the relative density of the TPMS structures was found to be highly correlated with their anti-vibration capacity and loading capacity. Superior anti-vibration capacities were observed in bionic structures than in strut nodes, and increasing the relative density for the same bionic topology led to higher resistance to dynamic excitations. In dynamic application scenarios, for TPMS structures, the design with a lower relative density tended to be more cost-effective because it could withstand relatively greater vibration with a lighter weight. In addition, the TPMS structures were vibration capacity dominated at lower relative densities and loading capacity dominated at higher relative densities. These two observations may help designers with the balance of the two types of capacities during the design and testing.

## Figures and Tables

**Table 1:** Material properties of the SLA resin material.

Young’s Modulus (GPa)	Poisson’s Ratio	Density (g/mm^3^)	Yield Strength (MPa)	Ultimate Tensile Strength (MPa)
2.51	0.23	1.10	38.30	61.12

**Table 2:** Dimensions of the BCC RVE model.

*n*	*w* (mm)	*d* (mm)	*h* (mm)	*e* (mm)	$\bar{\rho }$
2	6	6	6	1.2	0.56

**Table 3:** Dimensions of the macroscopic 6×7×15 BCC model.

*w* (mm)	*d* (mm)	*h* (mm)	*e* (mm)	$\bar{\rho }$
21	18	45	1.2	0.56

**Table 4:** The natural frequency for the 6×7×15 macroscopic lattice and its homogeneous models with errors.

Mode	Natural Frequency (Hz)		
	BCC	Homogenization	|Error|
1	1152.9	1170.7	1.5205%
2	1312.7	1330.6	1.3453%
3	3675.0	3634.2	1.1227%
4	4764.7	4834.6	1.4458%
5	5312.2	5358.0	0.8548%
6	5658.2	5697.8	0.6950%

**Table 5:** Dimensions of the 6×6×6 BCC and TPMS models with controlled relative densities.

*n*	*w* (mm)	*d* (mm)	*h* (mm)	$\bar{\rho }$
6	18	18	18	0.56

## Data Availability

Data are contained within the article.
